# A Mixture Model of Truncated Zeta Distributions with Applications to Scientific Collaboration Networks †

**DOI:** 10.3390/e23050502

**Published:** 2021-04-22

**Authors:** Hohyun Jung, Frederick Kin Hing Phoa

**Affiliations:** 1 Institute of Statistical Science, Academia Sinica, Taipei City 11529, Taiwan; hhjung@stat.sinica.edu.tw; 2 Department of Statistics, Sungshin Women’s University, Seoul 02844, Korea

**Keywords:** truncated zeta distribution, power-law, collaboration network, degree distribution, mixture model

## Abstract

The degree distribution has attracted considerable attention from network scientists in the last few decades to have knowledge of the topological structure of networks. It is widely acknowledged that many real networks have power-law degree distributions. However, the deviation from such a behavior often appears when the range of degrees is small. Even worse, the conventional employment of the continuous power-law distribution usually causes an inaccurate inference as the degree should be discrete-valued. To remedy these obstacles, we propose a finite mixture model of truncated zeta distributions for a broad range of degrees that disobeys a power-law behavior in the range of small degrees while maintaining the scale-free behavior. The maximum likelihood algorithm alongside the model selection method is presented to estimate model parameters and the number of mixture components. The validity of the suggested algorithm is evidenced by Monte Carlo simulations. We apply our method to five disciplines of scientific collaboration networks with remarkable interpretations. The proposed model outperforms the other alternatives in terms of the goodness-of-fit.

## 1. Introduction

Network science focuses on the study of complex networks such as telecommunication, computer, biological, cognitive, and social networks. A network consists of nodes and links. The topological structure, which explores how nodes are connected in the system, has been investigated with great interest [1,2,3]. Network researchers have examined foundational network topologies using various network-related quantities such as the degree distribution, clustering coefficient, and average path length. Most networks are dynamic, so accordingly, network-related quantities also change over time. Studying the evolution of these network quantities could provide insight into the behavior of individuals expressed by nodes and the change of topological properties of the network. The evolution of a network has been studied from various perspectives, e.g., the community [4,5], rich-get-richer [1], node heterogeneity [2,6], and link persistence [3].

The degree of a node is the number of links connected to a node. The degree distribution of a network, P(k), tells us the probability of degree *k* that a randomly chosen node will have. One challenge in studying network science is to develop simplified measures that capture the network structure. The degree distribution is one of such measures that help to find influential nodes in the system. Many attempts have been made to study the degree distribution using Poisson, exponential, and power-law distributions. In particular, the analysis of the power-law degree distribution has been considered as one of the basic steps, and networks that have power-law degree distributions are often referred to as *scale-free* networks. We can frequently observe power-law degree distributions in collaboration, World Wide Web, protein–protein interactions, and semantic networks [7,8,9,10]. The emergence of hubs (highly connected nodes) is a consequence of a scale-free property of networks. A rich-get-richer mechanism, also called a popularity effect [11], has been known to produce power-law degree distributions and hubs [1,6].

Many dynamic network models have been developed to explain the power-law degree distribution in real networks. The most widely known dynamic model for the power-law degree distribution is the rich-get-richer generative model in Barabasi and Albert (1999) [1], called the BA model. They employ the preferential attachment mechanism in which nodes with more neighbors tend to receive more links from other nodes. Specifically, the algorithm of the BA model has the following parts with the parameter *m* that controls the number of links over time:

- Growth: At each time point, a node enters the network. Then the node tries to connect with *m* nodes in the network.
- Preferential attachment: The newly entered node connects with node *i* with probability proportional to the degree of node *i*.

The BA model yields a power-law degree distribution with exponent 3, i.e., $P\left(k\right)\propto k^{-3}$. The exact form, given by Bollobas et al. in [12], is:

(1) $$\begin{array}{c} P\left(k|m\right)=\frac{2m(m+1)}{k(k+1)(k+2)},\;k=m,m+1,\cdots . \end{array}$$

There are many variant models to cover a broad range of power-law exponents [13,14,15], degree correlations [16], accelerating growth [17,18,19], and node heterogeneity [6,20,21]. Another model for generating the power-law degree distribution is a copying network model presented in Kumar et al. (2000) [22]. In this model, newly entered nodes randomly select some existing nodes and copy some of the links.

The power-law distribution has the form of $P\left(k\right)\propto k^{-\alpha }$, which can be expressed as lnP(k)=−αlnk+constant. Therefore, a straight line of lnP(k) plot on lnk (log-log plot) can be an indication of a power-law relationship, and its slope is a power-law exponent. In real networks, however, the degree distribution does not have a shape of a straight line in the entire range. There are many empirical distributions where the power-law behavior is not observed in the range of small degrees. Many variants of the power-law distribution are developed to address this issue, such as generalized power-law distributions [23,24], composite distributions with threshold [25,26], and power-law distributions with an exponential cutoff [27,28]. However, these methods do not consider the essential foundation of the power-law. According to the BA model and its variants, the power-law nature is an inherent property exhibited from the preferential attachment rule. The model presented in this paper preserves the power-law nature to avoid manual modifications of the power-law distribution function, given by $P\left(k\right)\propto k^{-\alpha }$.

Note that the BA model has a parameter *m*. Jordan (2006) [29] relieved the constant *m* condition that, at each time, the number *M* of connections can change over time according to the distribution of *M*. The degree distribution turned out to be

(2) $$\begin{array}{c} P\left(k\right)=\frac{2E[M(M+1)1(M\leq k)]}{k(k+1)(k+2)},\;k=1,2,\cdots .\; \end{array}$$

We note the following statistical property.

**Theorem** **1.** *Suppose that M has a finite support, $M=1,\cdots ,M_{max}$. Then the degree distribution of Jordan’s model can be expressed as a mixture distribution, given by*

(3) $$\begin{array}{c} P\left(k\right)=\sum_{m=1}^{M_{max}}w_{m}P\left(k|m\right),\;k=1,2,\cdots , \end{array}$$

*where $w_{m}=P\left(M=m\right)$ is a mixture weight corresponding to the mth mixture component P(k|m) in Equation (1), $m=1,\cdots ,M_{max}$.*

**Proof.**  Note that we can rewrite P(k|m) and P(k) as

$$\begin{array}{cc} P(k|m) & =\frac{2m(m+1)}{k(k+1)(k+2)}1\left(k\geq m\right)\\ P(k) & =\sum_{m=1}^{M_{max}}w_{m}\frac{2m(m+1)}{k(k+1)(k+2)}1\left(m\leq k\right) \end{array}$$

from Equations (1) and (2). Then, we have

$$\begin{array}{cc} P(k) & =\sum_{m=1}^{M_{max}}w_{m}\frac{2m(m+1)}{k(k+1)(k+2)}1\left(m\leq k\right)\\ \; & =\sum_{m=1}^{M_{max}}w_{m}P\left(k|m\right) \end{array}$$

by 𝟙(k≥m)=𝟙(m≤k). □

Theorem 1 suggests that the degree distribution might be expressed as a mixture distribution. For example, we consider a network where a new node connects to one or two existing nodes, with probabilities P(M=1)=2/3 and P(M=2)=1/3. Then we have the following degree distribution in a mixture form of the two BA model’s distributions with different *m*,

$$\begin{array}{c} P\left(k\right)=\frac{2}{3}P\left(k|m=1\right)+\frac{1}{3}P\left(k|m=2\right). \end{array}$$

Inspired by this property, we consider a mixture model as an explanation of the deviation from the power-law in the range of small degrees.

Moreover, many studies have considered the degree as a continuous variable using continuity assumptions [30,31]. This approach may mislead researchers since the degree is discrete-valued. Therefore, a discrete power-law distribution, called a truncated zeta distribution, is used in this paper.

In this study, we propose a mixture model of truncated zeta distributions for the analysis of degree distributions. The proposed model covers the entire range of the degree distribution through a mixture of truncated zeta distributions while maintaining the scale-free nature of a network. We can characterize the degree distribution more accurately through the discrete version of the power-law distribution. In addition, we present the maximum likelihood estimation algorithm along with a model selection method. A simulation study examines the validity of the proposed estimation procedure. In addition, real collaboration networks are investigated with the proposed model to describe the characteristics of the degree distribution.

We focus on analyzing actual scientific collaboration networks and have made significant advancements compared to the previous work in Jung and Phoa (2020) [32]. The major improvements are as follows:

- We detected some inconsistency in the scientific collaboration data. For example, “Smith, James,” “Smith, John,” and “Smith, Jacob” are all stated as “Smith, J” before 2007. Hence, we change the period of data to avoid the author name inconsistency for accurate inferences.
- Moreover, a more elaborate analysis of the real data is conducted with noteworthy interpretations.
- The validity of the presented algorithm is addressed with Monte Carlo simulations.
- Extensive comparison studies are performed to show the superiority of the proposed model. We compared the proposed model with generalized Pareto models as well as base models that lack discreteness or mixture nature.
- We provide more detailed explanations throughout the paper.

The rest of the paper is organized as follows. The continuous and discrete power-law distributions are defined in Section 2, and the proposed mixture model of truncated zeta distributions is defined in Section 3. Section 4 presents the estimation method of the mixture model, and the validity of the estimation procedure is demonstrated in Section 5. In Section 6, we analyze the scientific collaboration network by applying the proposed mixture model with interpretations. Section 7 concludes the paper.

## 2. The Power-Law Distribution

### 2.1. Continuous Power-Law Distribution

The probability density function (pdf) of the continuous power-law distribution parameterized by the power-law exponent α>1 and the minimum value l>0, denoted by PL(α,l), is given by [7]

(4) $$\begin{array}{c} f\left(x|\alpha ,l\right)=\left(\alpha -1\right)l^{\alpha -1}x^{-\alpha },\;x\geq l. \end{array}$$

Note that the support is real values greater than or equal to *l*.

The complementary cumulative distribution function is useful to describe the tail of the power-law distribution, expressed as

$$\begin{array}{c} P\left(X\geq x\right)={\left(\frac{x}{l}\right)}^{-\alpha +1},\;x\geq l. \end{array}$$

Its moments are given by

$$\begin{array}{c} E\left[X^{m}\right]=\frac{\alpha -1}{\alpha -1-m}l^{m}, \end{array}$$

provided α>m+1. We can deduce that PL(α,l) has the mean and variance given by

$$\begin{array}{c} E\left[X\right]=\frac{\alpha -1}{\alpha -2}l,\;Var\left[X\right]=\frac{\alpha -1}{\left(\alpha -3\right){\left(\alpha -2\right)}^{2}}l^{2}, \end{array}$$

where the mean and variance are well-defined for α>2 and α>3, respectively.

The continuous power-law distribution has been widely used in the analysis of the degree distribution. To analyze the discrete-valued variable degree, we need an approximation method. Setting a constant *c*, 0≤c≤1, for the correction of continuity, the degree distribution can be approximated by

(5) $$\begin{array}{c} f\left(k|\alpha ,l\right)\approx \int_{k-c}^{k+1-c}f\left(x|\alpha ,l\right)dx \end{array}$$

for an integer value *k*.

One of the most common approaches is to round values to the nearest integer, which corresponds to c=0.5 [7,33]. This *rounding* approach is acceptable when considering the tail part of the power-law distribution. However, if *k* is small, the constant *c* that satisfies the exact equation of (5) may be considerably less than 0.5, and it should be avoided. According to Clauset et al. (2009) in [7], the rounding approach is reasonable for k>6.

Since the node degree is usually small, the approximation may lead to misunderstanding when performing statistical analysis on the degree distribution, such as generating node degrees and fitting the distribution to real data. We consider the *exact* version of the discrete power-law in the next subsection.

### 2.2. Truncated Zeta Distribution

The truncated zeta distribution, denoted by TZ(α,l), is a discrete form of the power-law distribution. Parameters are the same as the continuous power-law distribution in which α>1 is the power-law exponent and l>0 is the minimum value. The probability mass function (pmf) of TZ(α,l) is given by [7]

(6) $$\begin{array}{c} g\left(k|\alpha ,l\right)=\frac{1}{\zeta (\alpha ,l)}k^{-\alpha },\;k=l,l+1,\cdots , \end{array}$$

where ζ(·,·) is the Hurwitz zeta function

$$\begin{array}{c} \zeta \left(\alpha ,l\right)=\sum_{k=l}^{\infty }k^{-\alpha },\; \end{array}$$

which can be regarded as the normalizing constant of the distribution. The Hurwitz zeta function in Equation (6) corresponds to the continuous counterpart $1/\left(\alpha -1\right)l^{\alpha -1}$ in Equation (4) via the upper Riemann sum approximation, expressed as

$$\begin{array}{c} \zeta \left(\alpha ,l\right)≳\int_{l}^{\infty }x^{-\alpha }dx=1/\left(\alpha -1\right)l^{\alpha -1}.\; \end{array}$$

The complementary cumulative distribution function is given by

$$\begin{array}{c} P\left(K\geq k\right)=\frac{\zeta (\alpha ,k)}{\zeta (\alpha ,l)},\;k\geq l. \end{array}$$

Next, the moments of the truncated zeta distribution are expressed as

$$\begin{array}{c} E\left[K^{m}\right]=\frac{\zeta (\alpha -m,l)}{\zeta (\alpha ,l)},\;\alpha >m+1. \end{array}$$

We can straightforwardly derive the mean and variance as

$$\begin{array}{c} E\left[X\right]=\frac{\zeta (\alpha -1,l)}{\zeta (\alpha ,l)},\;Var\left[X\right]=\frac{\zeta \left(\alpha ,l\right)\zeta \left(\alpha -2,l\right)-\zeta^{2}\left(\alpha -1,l\right)}{\zeta^{2}\left(\alpha ,l\right)}, \end{array}$$

provided α>2 for the mean and α>3 for the variance.

**Figure 1:**
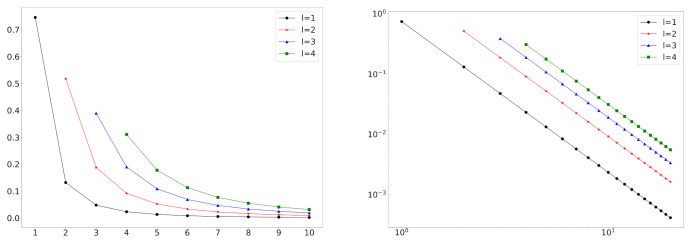
The pmfs of TZ(α,l) on a standard scale (**left**) and a log-log scale (**right**) when α=2.50 and l=1,2,3,4.

Figure 1 shows some pmfs of TZ(α,l) when α=2.50. We can check that the straight line of a log-log plot can be strong evidence of a power-law behavior.

## 3. Truncated Zeta Mixture Model

We consider the finite mixture model of truncated zeta distributions by fixing the power-law exponent α for mixture components while varying minimum values to produce a mixture of truncated zeta distributions. The probability mass function is represented as

(7) $$\begin{array}{c} p\left(k|\alpha ,L,w\right)=\sum_{l=1}^{L}w_{l}g\left(k|\alpha ,l\right),\;k=1,2,\cdots , \end{array}$$

where g(k|α,l) is the pmf of TZ(α,l), and *L* is the number of mixture components. Mixture weights $w=(w_{1},w_{2},\cdots ,w_{L})$

$$\begin{array}{c} w_{l}\geq 0,\;l=1,2,\cdots ,L-1,\;w_{L}>0,\;and\;\sum_{l=1}^{L}w_{l}=1. \end{array}$$

In this paper, we assume that the minimum value *l* is equal to 1, but it can be modified according to the data. The tail of most real networks follows the power-law distribution, and Equation (7) has the exact power-law behavior for sufficiently large degrees.

**Theorem** **2.** *For k larger than or equal to L, the truncated zeta mixture distribution in Equation (7) has the exact power-law relationship, given by*

$$\begin{array}{c} p\left(k|\alpha ,L,w\right)\propto k^{-\alpha }. \end{array}$$

**Proof.**  By using the pmf of TZ(α,l), we can write

$$\begin{array}{c} p\left(k|\alpha ,L,w\right)=\left(\sum_{l=1}^{L}\frac{w_{l}}{\zeta (\alpha ,l)}\right)k^{-\alpha }. \end{array}$$

Since the term inside the bracket is independent with *k*, the pmf of the mixture is proportional to $k^{-\alpha }$. □

Mixture models may suffer from the non-identifiability issue even for finite mixtures. The following theorem proves that the proposed truncated zeta mixture model is identifiable.

**Theorem** **3.** *The mixture distribution Equation (7) is identifiable with respect to α, L, and w.*

**Proof.**  Let $p(k|\alpha_{1},L_{1},w_{1})$ and $p(k|\alpha_{2},L_{2},w_{2})$ be the mixture distributions of $\alpha_{1}$, $L_{1}$, $w_{1}=\left(w_{11},w_{12},\cdots ,w_{1L}\right)$ and $\alpha_{2}$, $L_{2}$, $w_{2}=\left(w_{21},w_{22},\cdots ,w_{2L}\right)$, respectively. Suppose that $p(k|\alpha_{1},L_{1},w_{1})$ and $p(k|\alpha_{2},L_{2},w_{2})$ are identical, i.e., $p\left(k|\alpha_{1},L_{1},w_{1}\right)=p\left(k|\alpha_{2},L_{2},w_{2}\right)$ for all k=1,2,⋯. Further, we define the slope function s(k|α,L,w) of the log-log degree distribution, given by

$$\begin{array}{c} s\left(k|\alpha ,L,w\right)=\frac{\ln p(k+1|\alpha ,L,w)-\ln p(k|\alpha ,L,w)}{\ln(k+1)-\ln(k)},\;k=1,2,\cdots . \end{array}$$

The equality of the two mixture distributions give the identical slope function, $s\left(k|\alpha_{1},L_{1},w_{1}\right)=s\left(k|\alpha_{2},L_{2},w_{2}\right)$ for all k=1,2,⋯. We then have α=s(k|α,L,w) for the sufficiently large *k*$\left(\geq \max\left\{L_{1},L_{2}\right\}\right)$. Therefore, we obtain $\alpha_{1}=\alpha_{2}$. The number of mixture components *L* is the largest integer *k* such that s(k−1|α,L,w)≠α, and we also have $L_{1}=L_{2}$. Let *L*$\left(=L_{1}=L_{2}\right)$ and α$\left(=\alpha_{1}=\alpha_{2}\right)$ be the common number of mixture components and the power-law exponent.

Using $p\left(k|\alpha_{1},L_{1},w_{1}\right)=p\left(k|\alpha_{2},L_{2},w_{2}\right)$ for k=1,2,⋯,L, we have the following *L* equations:

$$\begin{array}{cc} w_{11}g\left(1|\alpha ,1\right) & =w_{21}g\left(1|\alpha ,1\right),\\ w_{11}g\left(2|\alpha ,1\right)+w_{12}g\left(2|\alpha ,2\right) & =w_{21}g\left(2|\alpha ,1\right)+w_{22}g\left(2|\alpha ,2\right),\\ \vdots \\ w_{11}g\left(L|\alpha ,1\right)+\cdots +w_{1L}g\left(L|\alpha ,L\right) & =w_{21}g\left(L|\alpha ,1\right)+\cdots +w_{2L}g\left(L|\alpha ,L\right). \end{array}$$

By solving these equations, we get $w_{1l}=w_{2l}$ for l=1,2,⋯,L. Thus, the mixture of truncated zeta distributions is identifiable. □

**Figure 2:**
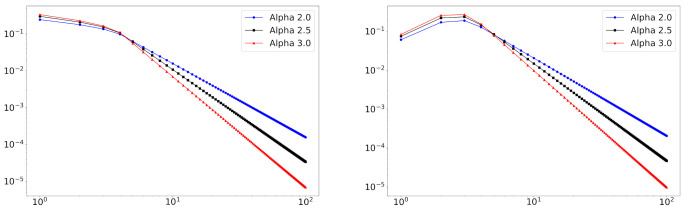
The pmfs of the mixture of truncated zeta distributions with L=4 on a log-log scale. Mixture weights are w=(0.4,0.3,0.2,0.1) (**left**) and w=(0.1,0.4,0.4,0.1) (**right**). Power-law exponents are α=2.0 (blue), α=2.5 (black), and α=3.0 (red).

In Figure 2, we depict some log-log plots of mixture distributions. We can see that this model can handle empirical degree distributions that do not follow the power-law distribution at small degrees.

## 4. Estimation Algorithm

We use the Expectation-Maximization (EM) algorithm to estimate the exponent parameter α and mixture weights *w* for a given number of mixture components *L*. Let $k=(k_{1},k_{2},\cdots ,k_{N})$ be the observed data, and $z_{n}$ be the *membership* of $k_{n}$, where the membership $z_{n}$ is assigned as $z_{n}=l$ if $k_{n}$ is from the *l*th mixture component TZ(α,l). We consider the membership variable $z=(z_{1},z_{2},\cdots ,z_{N})$ as missing. Let $\theta =\left(\alpha ,w\right)=\left(\alpha ,w_{1},\cdots ,w_{L}\right)$ be the parameters of the mixture model.

The complete-data likelihood function is given by

$$\begin{array}{cc} L(\theta |k,z) & =\prod_{n=1}^{N}p\left(z_{n}|w\right)g\left(k_{n}|\alpha ,z_{n}\right)\\ \; & =\prod_{n=1}^{N}w_{z_{n}}\frac{1}{\zeta (\alpha ,z_{n})}k_{n}^{-\alpha },\;k_{n}=z_{n},z_{n}+1,\cdots . \end{array}$$

We proceed by taking the logarithms of both sides to have the complete-data log-likelihood function:

$$\begin{array}{c} \ln L\left(\theta |k,z\right)=\sum_{n=1}^{N}\left(\ln w_{z_{n}}-\ln\zeta \left(\alpha ,z_{n}\right)-\alpha \ln k_{n}\right).\; \end{array}$$

We define $Q(\theta |\theta^{*})$ as the expected value of the log-likelihood given the observed data *k* and the current parameter estimate $\theta^{*}=\left(\alpha^{*},w^{*}\right)$, which can be expressed as

$$\begin{array}{cc} Q(\theta |\theta^{*}) & =E\left[\ln L\left(\theta |k,Z\right)|k,\theta^{*}\right]\\ \; & =\sum_{n=1}^{N}\sum_{l=1}^{L}\gamma \left(l,n,\theta^{*}\right)\left(\ln w_{l}-\ln\zeta \left(\alpha ,l\right)-\alpha \ln k_{n}\right), \end{array}$$

where $\gamma (l,n,\theta^{*})$ is the *membership responsibility* of the *n*th observation $k_{n}$ corresponding to the *l*th mixture component TZ(α,l). They are defined by the posterior probabilities of mixture component memberships for each observation,

(8) $$\begin{array}{cc} \gamma (l,n,\theta^{*}) & =P(z_{n}=l|k_{n},\theta^{*})\\ \; & =\frac{w_{l}^{*}g\left(k_{n}|\alpha^{*},l\right)}{\sum_{l^{\prime }=1}^{L}w_{l^{\prime }}^{*}g\left(k_{n}|\alpha^{*},l^{\prime }\right)},\;l=1,2,\cdots ,L,\;n=1,2,\cdots ,N. \end{array}$$

The E-step computes membership responsibilities.

In the M-step, a new parameter estimate $\overset{ˇ}{\theta }={\mathrm{argmax}}_{\theta }Q\left(\theta |\theta^{*}\right)$ is computed using the quantity previously computed in the E-step. To find $\overset{ˇ}{w}$, we need to solve the following optimization problem:

$$\begin{array}{cc} Maximize & \;\sum_{n=1}^{N}\sum_{l=1}^{L}\gamma \left(l,n,\theta^{*}\right)\ln w_{l},\\ Subjectto & \;\sum_{l=1}^{L}w_{l}=1,\;and\;w_{l}\geq 0,\;l=1,2,\cdots ,L. \end{array}$$

The Lagrange multiplier method yields

(9) $$\begin{array}{c} {\overset{ˇ}{w}}_{l}=\frac{1}{N}\sum_{n=1}^{N}\gamma \left(l,n,\theta^{*}\right),\;l=1,2,\cdots ,L. \end{array}$$

Next, $\overset{ˇ}{\alpha }$ can be found in the partial derivative of *Q* with respect to α, given by

$$\begin{array}{c} \frac{\partial Q(\theta |\theta^{*})}{\partial \alpha }=-\sum_{n=1}^{N}\sum_{l=1}^{L}\gamma \left(l,n,\theta^{*}\right)\left(\frac{\zeta_{\alpha }\left(\alpha ,l\right)}{\zeta (\alpha ,l)}+\ln k_{n}\right). \end{array}$$

Here, $\zeta_{\alpha }\left(\alpha ,l\right)$ is the partial derivative of the Hurwitz zeta function with respect to α, given by

$$\begin{array}{c} \zeta_{\alpha }\left(\alpha ,l\right)=\frac{\partial \zeta (\alpha ,l)}{\partial \alpha }=-\sum_{k=l}^{\infty }\left(\ln k\right)k^{-\alpha }. \end{array}$$

Then, the equation

(10) $$\begin{array}{c} \frac{\partial Q(\theta |\theta^{*})}{\partial \alpha }=0 \end{array}$$

gives the desired $\overset{ˇ}{\alpha }$. Unfortunately, a closed-form solution does not exist in general. In this paper, we employ Brent’s method [34] to solve Equation (10) with respect to α.

The two steps are necessarily repeated until the convergence obtains the final parameter estimate $\hat{\theta }$. The process is summarized in Algorithm 1.

In order to select the number of mixture components *L*, we employ the Bayesian information criterion (BIC) considering the trade-off between the goodness-of-fit and the complexity of the model. BIC is given by the following formula:

$$\begin{array}{c} BIC=L\ln N-2\sum_{n=1}^{N}\ln p\left(k_{n}|\alpha ,L,w\right), \end{array}$$

where α and *w* are the obtained estimated parameters given the number of mixture components *L*. We choose *L* giving the smallest BIC, where the candidates of *L* are the integers from 1 to the minimum of the two values: 100 and the nearest integer to 0.90×(maximumdegree).
**Algorithm 1:** EM Algorithm
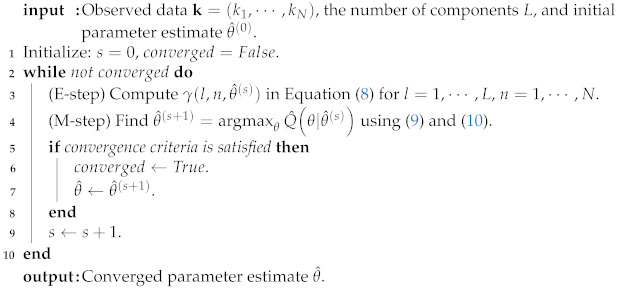


## 5. Monte Carlo Simulation

We study the validity of the presented estimation methods using the synthetic data. We consider three values of true power-law exponents α=2.50,3.00,3.50 and also three values of the number of mixture components L=2,4,6. For each pair of α and *L*, we generate 1000 samples from the finite mixture of truncated zeta distributions in Equation (7). Each process is repeated 30 times. Mixture weights $w_{l}$, l=1,2,⋯,L are set to 1/L for each dataset.

We assume that the true number of components is given. Algorithm 1 is applied to each dataset to obtain the parameter estimate $\hat{\theta }=\left(\hat{\alpha },\hat{w}\right)$. Table 1 presents the parameter estimation result. We find that the average values of estimated parameters are very close to the true parameters, implying that the EM algorithm works well in estimating the exponent parameter α and weights *w*.

Next, we assume that the number *L* of mixture components is not given. We will check whether the presented algorithm can find the appropriate number of mixture components. Note that BIC is used as a model selection criterion for the generated datasets. Table 2 shows the result of determining *L*. The result indicates that the maximum likelihood method yields a reasonable estimation result in finding *L*.

## 6. Application: Collaboration Networks

### 6.1. The Data

We study the scientific collaboration network obtained from the Web of Science, where a large-scale database collects the information of all published scientific articles in the world. Among all 275 disciplines, we randomly choose five, which are Biotechnology and Applied Microbiology, Computer Science, Environmental Science, Materials Science, and Physical Chemistry, for demonstration. We reorganize the Web of Science database into a network data structure such that the nodes of networks are authors, and two authors are connected by an undirected link if there is at least one paper co-authored by them. We employ the data from 2007 to 2016 as the inconsistency of author’s names is observed before the year 2007. The data from 2007 to 2009 are used to accumulate data so that the dependence of node degrees can be ignorable. We analyze the degree distribution of the data from 2010 to 2016. Table 3 shows the summary statistics in the year 2016.

### 6.2. Application of the Truncated Zeta Mixture Model

We apply the presented estimation method to degree distributions from 2010 to 2016.

**Figure 3:**
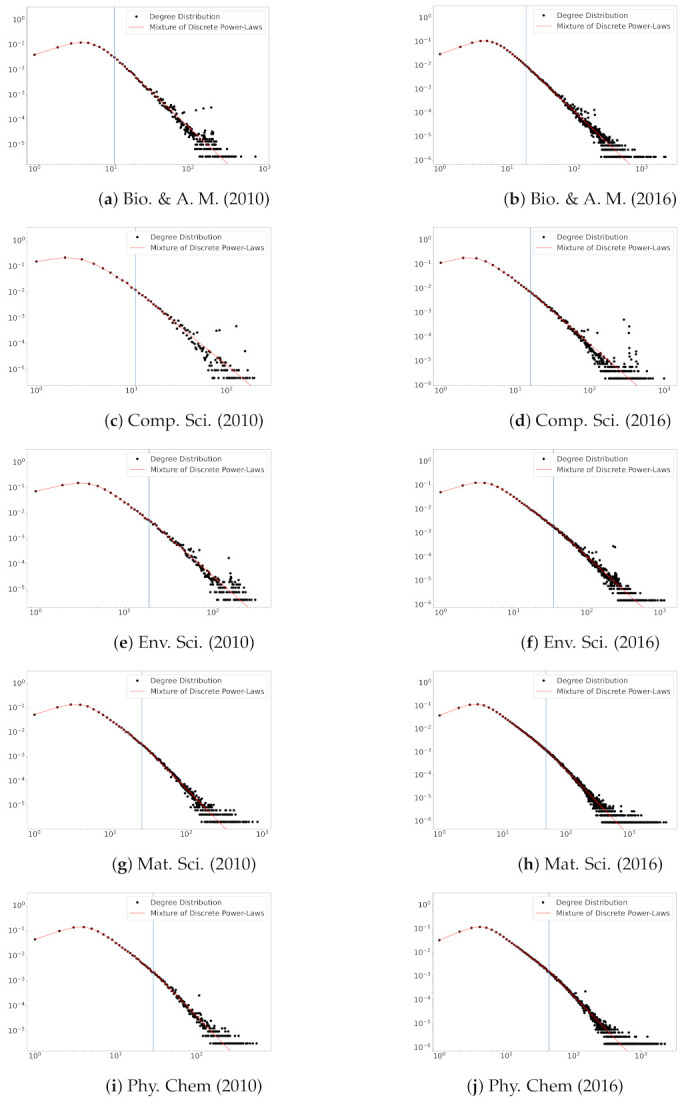
Degree distributions and fitted curves for five fields of scientific collaboration in the years 2010 and 2016. The estimated *L* values are depicted in blue vertical lines.

In Figure 3, we plot degree distributions and the estimated fitting lines of the proposed model in the years 2001 and 2016. The plots suggest that the truncated zeta mixture shows a good performance in fitting degree distributions. The beginning points (see the vertical lines in Figure 3) of power-law behaviors are reasonably estimated via $\hat{L}$. This result shows the usefulness of the proposed model for the degree distribution that deviates from the power-law distribution on a small degree part.

**Figure 4:**
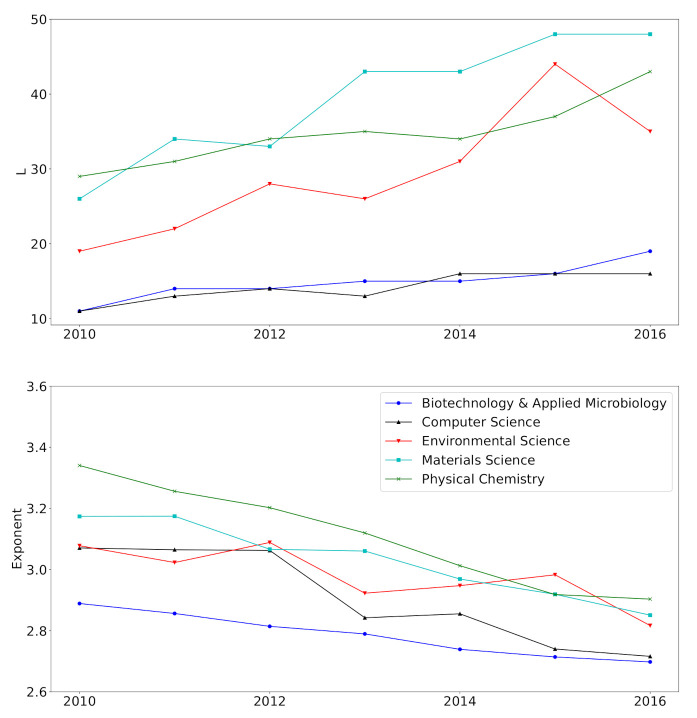
The change of the estimated number of mixture components *L* (**top**) and power-law exponents α (**bottom**).

Figure 4 shows the temporal changes of $\hat{L}$ and $\hat{\alpha }$. The estimated number *L* of mixture components exhibits a rising tendency, and $\hat{\alpha }$ shows an opposite trend. They constantly move towards large *L* and small α regions for all fields, indicating that the stabilization of the degree distribution has not yet been achieved. Many network scientists [1,13,14] have concentrated on the stationary or the converged degree distribution. The result implies that, however, non-stationary network models are of great importance, such as acceleration models [15]. Moreover, many works in real data analysis have focused on the power-law exponent of a snapshot of a network. However, the temporal variation of $\hat{\alpha }$ tells us that the significance of temporal models can account for the temporal change of the power-law exponent.

It should be noted that $\hat{L}$ tends to approach different values across fields. According to Equation (3) and the relevant interpretation in Section 1, the number of mixture components *L* and mixture weights *w* are closely related to the distribution of the number of links in the system. Therefore, $\hat{L}$ could be a measure to the distribution of the number of links. On the other hand, $\hat{\alpha }$ seems to converge a value near 2.80 for all fields, suggesting that α could be a network-specific quantity instead of a field-specific quantity.

We now focus on the field of Computer Science. Table 4 presents the result of applying the model to the field of Computer Science in more detail. We can observe an interesting pattern in the estimated mixture weights, where ${\hat{w}}_{1}$ and ${\hat{w}}_{2}$ show decreasing trends whilst ${\hat{w}}_{3}$, ${\hat{w}}_{4}$, and ${\hat{w}}_{5}$ show the opposite. According to Jordan’s model [29] and Equation (3), mixture weights have much to do with the number of newly made links. The decreasing trend of $w_{1}$ and $w_{2}$ and the increasing trend of $w_{3}$, $w_{4}$, ⋯ indicates the increasing number of links over time. As shown in Table 4, the number of created links tends to increase over time. Since there are many new links compared to new nodes (authors), the average degree gradually increased from 4.70 in 2010 to 6.68 in 2016. The increase in the average degree also explains that the state of this network is still evolving. With the rapid advancement in technology and science, many publications have been produced by researchers. In particular, Computer Science is making greater progress due to recently emerging areas: artificial intelligence and big data. The proposed model tries to explain the increasing mean degree in two ways: decreasing power-law exponents α and an appropriate change in the mixture weight, suggesting that the proposed model is helpful to describe the change of the network pattern.

### 6.3. Comparison to Other Models

Our model is developed to deal with two essential characteristics of the degree distribution: the non-power-law behavior at small degrees and the discreteness. We study the superiority of the proposed model to base models that lack one of these characteristics.

**Figure 5:**
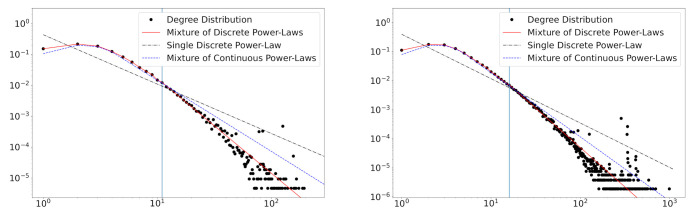
The degree distributions of the collaboration network data of the Computer Science field in the years 2010 (**left**) and 2016 (**right**). The estimated mixture of truncated zeta distributions (red solid), standard discrete power-law distributions (black dash-dot), and the mixture of continuous power-law distributions (blue dashed) are presented. Vertical lines refer to the estimated number $\hat{L}$ of mixture components.

We first consider the standard discrete power-law distribution, TZ(α,1). The estimates of the power-law exponent α are obtained by fitting the data into TZ(α,1), and the result is presented in Table 5. The $\hat{\alpha }$ estimates of the standard discrete power-law distribution are smaller than those of the mixture distribution. As we can observe in Figure 5, the small-degree data that deviates from the power-law makes the exponent small. The estimated fitting lines indicate that the standard discrete power-law distribution is inappropriate to describe the degree distribution.

Next, the degree distribution is applied to the mixture of the continuous power-law distribution using the rounding method with the constant c=0.5 of the continuity correction. The continuous version of the mixture of truncated zeta distributions can be constructed by the substitution of TZ(α,l) to PL(α,l−c). In addition, we imitate the method in Section 4 for the continuous mixture to compare the performance of the continuous mixture with the discrete mixture. The estimation procedure is identical to the case of the discrete mixture model. Table 5 presents the estimation result of α and mixture weights *w*. The estimates $\hat{L}$ and $\hat{\alpha }$ are much smaller in continuous mixture models due to the non-exactness of the continuous model. In addition, estimated mixture weights $\hat{w}$ are considerably different from the discrete mixture. According to Equation (3), mixture weights are involved in the distribution of the number of links. Therefore, we should use the discrete version of the power-law to determine mixture weights correctly. Figure 5 shows that the fitting lines of the continuous mixture seem to deviate from empirical distributions.

As we can see in Table 4 and Table 5, the smallest BIC values are achieved in the proposed model as well. Thus, we can conclude that the proposed model outperforms the continuous model as well as the standard discrete power-law model.

Next, we compare the proposed model with generalized Pareto distributions in which all degree ranges are covered. We use both continuous and discrete types of generalized Pareto distributions. The complementary cumulative density function of the continuous generalized Pareto distribution GPD(μ=0.5,σ,ξ) is given by

$$\begin{array}{c} P\left(X\geq x\right)={\left(1+\frac{\xi (x-\mu )}{\sigma }\right)}^{-1/\xi },\;x\geq \mu . \end{array}$$

**Figure 6:**
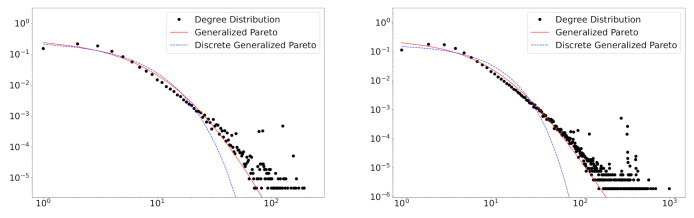
The degree distributions of the collaboration network data of the Computer Science field in the years 2010 (**left**) and 2016 (**right**). The estimated fitting lines of continuous (red solid) and discrete (blue dashed) generalized Pareto distributions are presented.

There are few studies concerning the discrete version of the generalized Pareto distribution. We here use the model in Prieto et al. (2013) [35], expressed as DGP(α,λ,μ=1). This distribution has advantages over the continuous distribution since the node degree is discrete. Table 6 shows the results of discrete and continuous generalized Pareto distributions applied to the field of Computer Science. In Figure 6, we plot fitting results in the years 2010 and 2016.

Interestingly, Figure 6 suggests that the continuous version has better fitting results. The discrete version has difficulty in explaining a range of large degrees. We can see that our model performs better than the two generalized Pareto distributions, as well indicated by the BIC values (see Table 4 and Table 6).

## 7. Concluding Remark

Inspired by Jordan’s model [29], a novel mixture model for the degree distribution is proposed to describe the entire range of degrees while maintaining the power-law or the scale-free property of a network. The truncated zeta distribution enables us to analyze discrete distributions for accuracy purposes. The parameter estimation procedure is presented along with the model selection criterion for determining the number of mixture components. A simulation study shows the validity of the suggested estimation procedure. The practical performance of the model is studied through the comparison analysis with the other techniques.

We perform the real data analysis on five disciplines of the scientific collaboration data obtained from the Web of Science. We observe the increasing tendency in the number of mixture components and the decreasing tendency in the power-law exponent. In addition, mixture weights change over time. It can be suggested from these results that the analyzed networks are still in an evolving state, highlighting the practical importance of non-stationary temporal network models. The non-convergence of the degree distribution might be due to the short-term analysis performed. Determining whether the collaboration network will stabilize the equilibrium remains as future work.

We can observe power-law distributions not only in the degree distribution but also in sandpile avalanches, species extinctions, city sizes, and so on. The proposed model could be useful when (i) the distribution does not follow the power-law only in small values while the power-law is suitable for large values, (ii) the background knowledge does not support the manual modification of the power-law relationship, or (iii) a mixture distribution can be regarded as reasonable for describing data.

## Figures and Tables

**Table 1:** Estimates of θ of the EM algorithm applied to the synthetic data with the true number of mixture components. We present the average (standard deviation in parentheses) values of estimated parameters $\hat{\theta }=\left(\hat{\alpha },\hat{w}\right)$ of 30 datasets for each case.

α	*L*	$w_{l}$	$\hat{\alpha }$	$\hat{w}$
2.50	2	0.50	$2.51_{\left(0.05\right)}$	$\left[0.49_{\left(0.02\right)},0.51_{\left(0.02\right)}\right]$
	4	0.25	$2.51_{\left(0.06\right)}$	$\left[0.25_{\left(0.01\right)},0.25_{\left(0.02\right)},0.25_{\left(0.03\right)},0.25_{\left(0.02\right)}\right]$
	6	0.17	$2.50_{\left(0.07\right)}$	$\left[0.17_{\left(0.02\right)},0.16_{\left(0.02\right)},0.17_{\left(0.03\right)},0.18_{\left(0.04\right)},0.16_{\left(0.04\right)},0.16_{\left(0.04\right)}\right]$
3.00	2	0.50	$3.01_{\left(0.09\right)}$	$\left[0.49_{\left(0.02\right)},0.51_{\left(0.02\right)}\right]$
	4	0.25	$3.03_{\left(0.10\right)}$	$\left[0.25_{\left(0.01\right)},0.24_{\left(0.02\right)},0.25_{\left(0.03\right)},0.26_{\left(0.03\right)}\right]$
	6	0.17	$3.03_{\left(0.11\right)}$	$\left[0.17_{\left(0.01\right)},0.17_{\left(0.02\right)},0.17_{\left(0.03\right)},0.17_{\left(0.03\right)},0.16_{\left(0.03\right)},0.17_{\left(0.03\right)}\right]$
3.50	2	0.50	$3.50_{\left(0.14\right)}$	$\left[0.50_{\left(0.02\right)},0.50_{\left(0.02\right)}\right]$
	4	0.25	$3.51_{\left(0.13\right)}$	$\left[0.25_{\left(0.02\right)},0.25_{\left(0.02\right)},0.26_{\left(0.02\right)},0.25_{\left(0.02\right)}\right]$
	6	0.17	$3.50_{\left(0.13\right)}$	$\left[0.17_{\left(0.01\right)},0.17_{\left(0.01\right)},0.17_{\left(0.02\right)},0.17_{\left(0.02\right)},0.15_{\left(0.03\right)},0.17_{\left(0.03\right)}\right]$

**Table 2:** The estimation result of *L* applied to the maximum likelihood method. $\hat{L}$ is selected with the smallest BIC for each dataset. The average, standard deviation, and count of $\hat{L}$ over 30 datasets are presented.

α	*L*	Average $\hat{L}$	St.Dev. $\hat{L}$	Count $\hat{L}$
2.50	2	2.03	0.18	{2: 29, 3: 1}
	4	4.00	0.00	{4: 30}
	6	5.87	0.34	{6: 26, 5: 4}
3.00	2	2.00	0.00	{2: 30}
	4	4.00	0.00	{4: 30}
	6	6.00	0.00	{6: 30}
3.50	2	2.00	0.00	{2: 30}
	4	4.00	0.00	{4: 30}
	6	5.97	0.18	{6: 29, 5: 1}

**Table 3:** The summary statistics of collaboration networks in the year 2016. For each field, we present the number of authors (nodes) and links between them. The mean, median, standard deviation, and maximum of degrees are also presented.

Field	Number of		Degree Distribution			
	Nodes	Links	Mean	Median	St.Dev.	Max.
Biotechnology & A. M.	729,478	3,977,919	10.91	7.00	19.52	2250
Computer Science	528,267	1,765,283	6.68	4.00	15.19	989
Environmental Science	680,924	3,291,780	9.67	5.00	17.42	1149
Materials Science	1,154,908	6,861,189	11.88	6.00	28.47	3801
Physical Chemistry	727,213	4,150,183	11.41	6.00	23.42	2260

**Table 4:** The results of $\hat{L}$, $\hat{\alpha }$, $\hat{w}$, and BIC using the proposed model for the discipline of Computer Science. We also present the number of new links and nodes as well as the mean degree over time.

Year	Discrete Mixture								Number of New		Mean Deg.
	$\hat{L}$	$\hat{\alpha }$	${\hat{w}}_{1}$	${\hat{w}}_{2}$	${\hat{w}}_{3}$	${\hat{w}}_{4}$	${\hat{w}}_{5}$	BIC	Nodes	Links	
2010	11	3.07	0.18	0.32	0.25	0.12	0.06	1,010,549	-	-	4.70
2011	13	3.06	0.17	0.30	0.25	0.12	0.06	1,241,555	43,237	132,560	4.94
2012	14	3.06	0.16	0.30	0.25	0.13	0.07	1,486,634	45,004	144,305	5.16
2013	13	2.84	0.16	0.30	0.26	0.13	0.07	1,813,101	56,392	256,646	5.78
2014	16	2.86	0.15	0.29	0.26	0.14	0.07	2,137,666	56,091	215,236	6.03
2015	16	2.74	0.15	0.29	0.26	0.14	0.07	2,481,780	57,317	277,983	6.48
2016	16	2.72	0.14	0.28	0.26	0.14	0.07	2,810,563	55,800	234,714	6.68

**Table 5:** The results of $\hat{L}$, $\hat{\alpha }$, $\hat{w}$, and BIC using the continuous mixture model for the discipline of Computer Science. Estimated α and BIC for the standard discrete power-law distribution TZ(α,1) are also presented.

Year	Continuous Mixture						Zeta	
	$\hat{L}$	$\hat{\alpha }$	${\hat{w}}_{1}$	${\hat{w}}_{2}$	${\hat{w}}_{3}$	BIC	$\hat{\alpha }$	BIC
2010	4	2.30	0.20	0.40	0.30	1,053,289	1.60	1,170,929
2011	4	2.29	0.19	0.39	0.30	1,290,015	1.59	1,436,579
2012	5	2.29	0.18	0.38	0.30	1,540,812	1.57	1,719,325
2013	5	2.26	0.17	0.37	0.30	1,871,783	1.56	2,090,369
2014	5	2.24	0.16	0.36	0.30	2,201,834	1.55	2,462,841
2015	5	2.22	0.16	0.35	0.31	2,550,130	1.54	2,852,831
2016	5	2.21	0.15	0.34	0.30	2,884,064	1.53	3,229,651

**Table 6:** The results of discrete and continuous generalized Pareto distributions applied to the field of Computer Science.

Year	Continuous Generalized Pareto			Discrete Generalized Pareto		
	$\hat{\sigma }$	$\hat{\xi }$	BIC	$\hat{\alpha }\;\left(10^{4}\right)$	$\hat{\lambda }\;\left(10^{-6}\right)$	BIC
2010	3.54	0.14	1,032,317	2.51	9.53	1,043,227
2011	3.68	0.16	1,267,627	5.42	4.17	1,282,398
2012	3.83	0.16	1,517,145	5.65	3.81	1,535,652
2013	3.93	0.21	1,852,799	5.69	3.34	1,911,678
2014	4.09	0.22	2,183,341	1.40	12.92	2,249,684
2015	4.20	0.25	2,535,774	5.63	2.98	2,633,601
2016	4.31	0.26	2,871,026	3.49	4.65	2,980,178

## Data Availability

Restrictions apply to the availability of these data. Data was obtained from a neo4j database refined from the Web of Science database. They are available from Dr. Frederick Kin Hing Phoa with the permission of the URA team of ISM (Japan) and Clarivate Analytics.
